# 

**Published:** 1896-08-01

**Authors:** 


"The standard of highest purity at present attainable."—*Lancet.*

CADBURY'S COCOA

NO ALKALIES USED.

### THE HOSPITAL

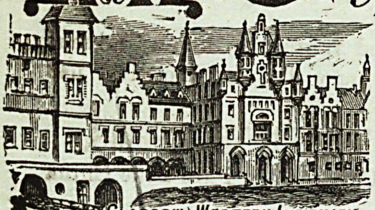

GLASGOW WESTERN INFIRMARY

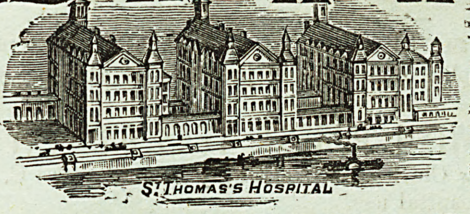

ST THOMAS'S HOSPITAL

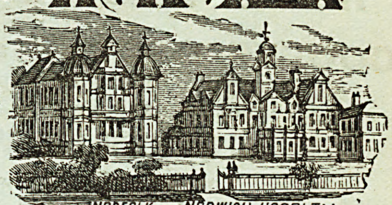

NORFOLK AND NORWICH HOSPITAL

No. 514. Vol. XX.

SATURDAY, AUGUST 1ST, 1896.

WITH EXTRA NURSING SUPPLEMENT.

[Registered at the General Post Office as a Newspaper for transmission in the United Kingdom and Abroad.]

